# Calcium Mass Balance during Citrate Hemodialysis: A Randomized Controlled Trial Comparing Normal and Low Ionized Calcium Target Ranges

**DOI:** 10.1371/journal.pone.0168593

**Published:** 2016-12-28

**Authors:** Jakob Gubensek, Alesa Orsag, Rafael Ponikvar, Jadranka Buturovic-Ponikvar

**Affiliations:** 1 Department of Nephrology, University Medical Centre Ljubljana, Ljubljana, Slovenia; 2 Faculty of Medicine, University of Ljubljana, Ljubljana, Slovenia; Medizinische Universitat Graz, AUSTRIA

## Abstract

**Background:**

Regional citrate anticoagulation (RCA) during hemodialysis interferes with calcium homeostasis. Optimal ionized calcium (iCa) target range during RCA and consequent calcium balance are unknown.

**Methods:**

In a randomized controlled trial (ACTRN12613001029785) 30 chronic hemodialysis patients were assigned to normal (1.1–1.2 mmol/) or low (0.95–1.05 mmol/l) iCa target range during a single hemodialysis with RCA. The primary outcome was calcium mass balance during the procedure, using a partial spent dialysate collection method; magnesium mass balance was also measured. Intact parathormone (iPTH), total calcium (tCa) and magnesium were measured before and after procedures.

**Results:**

Mean iCa during procedures was significantly different in the two groups (1.12±0.06 in normal and 1.06±0.07 mmol/l in low iCa group, p <0.001), resulting in different tCa (2.18±0.22 vs. 1.95±0.17, p = 0.003) after the procedure. Mean delivered calcium during the procedure was 58.3±4.8 mmol in the normal and 51.5±8.2 mmol in the low iCa group (p = 0.010), which resulted in a significantly higher mean positive calcium mass balance of 14.6±8.3 mmol (584±333 mg) per procedure in normal as compared to 7.2±8.5 mmol (290±341 mg) in low iCa group (p = 0.024). Linear mixed effects model showed a significant interaction effect of time and iCa target range group on iPTH, i.e. a significant increase in iPTH in the low as compared to normal iCa target group (p = 0.008). Magnesium mass balance was mildly negative and comparable in both groups.

**Conclusions:**

Low iCa target range resulted in a significantly less positive calcium mass balance, but in a significant increase in iPTH. To achieve a more neutral calcium balance, we recommend allowing a mild hypocalcemia during hemodialysis with RCA, especially when it is used for prolonged periods.

## Introduction

In recent years, regional citrate anticoagulation (RCA) has become the preferred method of anticoagulation in continuous renal replacement therapy [1, 2], while in chronic hemodialysis it is the preferred method in patients with increased bleeding risk. The main reason for the increased popularity of RCA is the excellent anticoagulation achieved, which is entirely regional, i.e., limited to the extracorporeal circuit, and enables better circuit run-times in continuous renal replacement therapy [2]. Furthermore, RCA is known to improve the biocompatibility of the hemodialysis procedure by reducing leukocyte [3, 4], platelet [4] and complement activation [5] in addition to the better inhibition of coagulation cascade, as compared to heparin anticoagulation [6]. The effects of citrate are mediated by severe hypocalcemia in the extracorporeal circuit, which reduces many of the calcium-dependant, blood-surface interactions. Currently, there are already some reports of small groups of chronic dialysis patients treated with long-term RCA hemodialysis due to persistently increased bleeding risk or other contraindications for heparin [7, 8]. In the future, if RCA becomes fully automated in intermittent hemodialysis [9, 10], as has been the case in continuous methods, and in light of its better biocompatibility, RCA may become the standard method of anticoagulation for all chronic hemodialysis patients [11].

When the prolonged use of RCA in a chronic hemodialysis patient is being contemplated, some additional aspects should be considered. Citrate binds calcium, which needs to be replaced during RCA to prevent hypocalcemia. But the exact ionized calcium (iCa) levels, maintained during citrate dialysis, also affect the calcium mass balance of the dialysis procedure. This topic has not yet been properly addressed in RCA, and the optimal ionized calcium target range to be maintained in a patient during citrate hemodialysis remains to be established. In heparin hemodialysis, the calcium mass balance resulting with the use of different dialysate calcium levels has been studied extensively [12–14]. Nevertheless, the optimal balance for the patient remains an area of controversy [15, 16]. In citrate dialysis, however, the calcium balance has not yet been precisely studied. While maintaining normal iCa during dialysis seems very reasonable and has been practiced by some groups [17, 18], allowing a mild hypocalcemia, the approach our group has taken [8, 19, 20] might compensate for the release of calcium from the citrate-calcium complexes occurring after the completion of dialysis as a result of citrate metabolism [18], thereby preventing an unwanted positive calcium balance [21].

The aim of the present randomized controlled study was to compare calcium mass balance with normal and low iCa target range during hemodialysis with RCA and to establish the optimal iCa target range.

## Subjects and Methods

### Study design

This was a two arm, parallel, open-label, single-center randomized controlled study, performed in a university hospital dialysis centre. We included in the study 30 adult chronic hemodialysis patients who required RCA due to increased bleeding risk between January—September 2014 and December 2015—January 2016; the study was paused in-between for organisational reasons. Patients were randomly assigned by the treating physician in accordance with a randomisation list in a 1:1 ratio to either a normal (1.1–1.2 mmol/) or a low (0.95–1.05 mmol/l) iCa target range during a single study hemodialysis procedure with RCA. The randomisation plan was obtained from the web site http://www.randomization.com/, using a method of randomly permuted blocks, the block size was two. Group allocation was done by the treating physician and concealment was achieved by sequentially numbered, sealed envelopes.

The sample size was determined with an on-line calculator (http://powerandsamplesize.com/Calculators/Compare-2-Means/2-Sample-Equality) based on the available data on a mean calcium balance of 5 mmol/session [18] with normal iCa target and assuming a 5 mmol standard deviation; an 80% power and a 5% alpha error was chosen to detect a 5 mmol between group difference using a one-sided t test. The sample size was rounded up to 15 patients in each group.

The study was conducted according to the principles expressed in the Declaration of Helsinki. All the patients signed a written informed consent prior to enrolment. The study was approved by the National Medical Ethics Committee (Ref. No. 89/03/13) and registered at the Australian New Zealand Clinical Trials Registry (ACTRN12613001029785).

### Hemodialysis procedure

During the hemodialysis procedures, we used single-use, high-flux dialyzers primed with saline before dialysis. Blood flow was set at 250 ml/min, and a calcium-free, magnesium 0.50 mmol/l dialysate was used at 500 ml/min. RCA was performed with a continuous infusion of 8% trisodium citrate (prepared by our hospital pharmacy) into the arterial line (at 150 ml/h) and 1 M calcium chloride into the venous line, starting at 13 ml/h in the low iCa group and at 15 ml/h in the normal iCa group. The calcium infusion rate was adjusted after each iCa measurement by 1–2 ml/h (in accordance with our clinical experience and without a pre-specified scheme) to achieve the desired iCa target range. iCa, taken from the arterial line of the dialysis circuit, was measured on an ABL800 Flex analyzer (Radiometer Medical, Bronshoj, Denmark) located at the Dialysis Centre; measurements were taken at the start of dialysis, after 30 minutes, after every full hour, and at the end of dialysis. The intact parathormone (iPTH) (Immulite 2000 Intact PTH, Siemens Healthcare, Erlangen, Germany), blood gas analysis (ABL800 Flex, see above), magnesium, total (tCa) and corrected calcium (cCa) were measured before and after dialysis using standard laboratory methods. Corrected calcium was calculated using the standard Payne formula: corrected calcium = serum calcium (mmol/l)—(0.025 x serum albumin (g/l)) + 1. As a safety secondary endpoint, the occurrence of hypocalcemia, defined as iCa < 0.90 mmol/l, was recorded. Visual assessment of the anticoagulation in the circuit was performed after dialysis by the dialysis nurse using a semi-quantitative score of 1 (worst) to 5 (best), as described previously [19].

### Calcium and magnesium mass balance

We used a partial spent dialysate collection method, which was shown to correlate very well with total spent dialysate collection [22]. An infusion pump was connected to the spent dialysate line to continuously collect spent dialysate at a rate of 300 ml/h, collecting a representative sample of approximately 1% of the total spent dialysate. The total spent dialysate volume was calculated by summing the volume of fresh dialysate prepared, which is recorded by the hemodialysis machine (Gambro AK 200 UltraS, Gambro, Lund, Sweden), the volume of gross ultrafiltration as programmed into the dialysis machine, and the volume of collected dialysate. As the volume of the collected dialysate sample is perceived by the dialysis machine as ultrafiltration, the actual desired ultrafiltration needs to be reduced by the volume of sampled dialysate. Samples of the partial spent dialysate were collected and stored at -20°C. Calcium and magnesium were measured on an Olympus AU400 analyser (Olympus, Tokyo, Japan). Calcium was measured using the O-cresolphthalein method; the coefficient of variation at the expected concentration range is 3.8%. It was shown that citrate in concentrations of up to 5 mmol/l (higher than what is expected in the blood in the extracorporeal circuit and therefore also in the dialysate) does not interfere with this method [23]. Magnesium was measured using the Xylidyl blue method, for which there is also evidence of non-interference with citrate in the expected concentration range [24]; the coefficient of variation is 1.2%.

The primary outcome measure was calcium mass balance during entire hemodialysis session, which was calculated as the difference between delivered calcium during dialysis (recorded in ml from the perfusor pump) and estimated calcium loss (calculated as measured dialysate calcium concentration x total spent dialysate volume). Magnesium mass balance, a secondary outcome, was also calculated as the difference between the spent dialysate magnesium concentration (measured) and fresh dialysate magnesium (i.e., 0.50 mmol/l), multiplied by the total spent dialysate volume. Magnesium was measured in a few samples of fresh dialysate and confirmed to be within ±0.01 mmol/l of the declared 0.50 mmol/l value for fresh dialysate.

### Statistical analysis

Data is presented as mean ± standard deviation (SD), median and interquartile range (IQR) or percentage, as appropriate. The changes in iPTH during a dialysis session were expressed as an absolute increase and as percent increase from the baseline. The calcium (primary outcome) and magnesium (secondary outcome) mass balance and other continuous variables were compared between the groups using the Student's T test. Since duration of hemodialysis was not equal for all patients, linear mixed effects regression analysis was used to test the relationship between two target iCa range groups and dependent variable. Two regression models were built, first with iCa values during dialysis and second with iPTH as dependent variable. Time, iCa target range group and interaction between the two were included as fixed effects and intercept was allowed to randomly vary in both models. Covariance structure was diagonal for the first and autoregressive for the second model. The frequency of hypocalcemia (a safety secondary outcome) was compared using the Yates corrected Chi-square test. A multiple linear regression model was constructed to test whether the variables, which were thought plausible to affect the calcium mass balance (i.e. ionized calcium before and after dialysis, dialysis duration and iPTH) in addition to the amount of infused calcium, were indeed independent predictors of calcium mass balance. Statistica 7.0 (StatSoft, Inc., Tulsa, USA) was used for statistical analysis and graphs, except for the mixed effects model, which was done with SPSS 23.0 (IBM, New York, USA).

## Results

**Fig 1:**
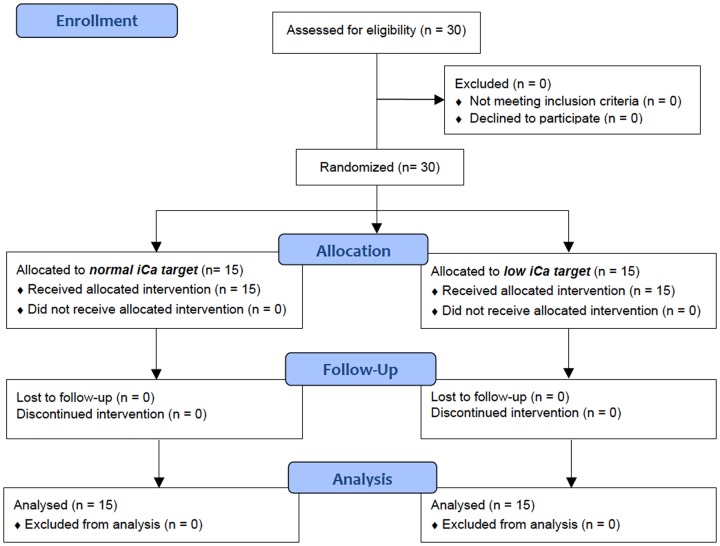
CONSORT flow diagram of the study.

**Fig 2:**
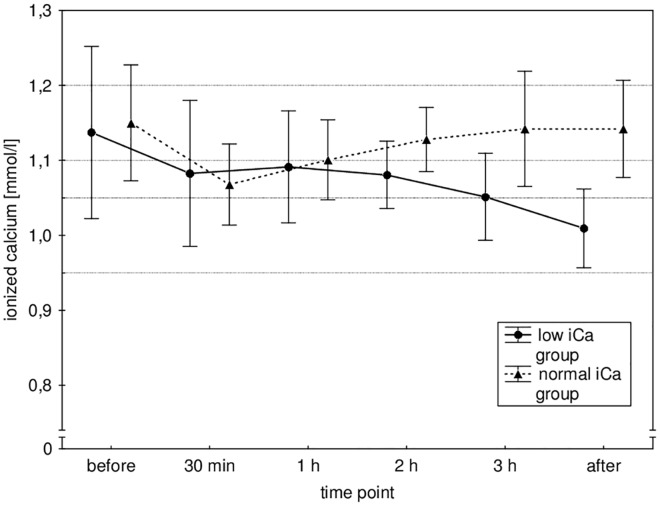
Patients' ionized calcium (iCa), measured from the arterial line, during the procedures in both iCa target groups. Data is shown as mean ± SD, both target ranges are marked with a dotted line.

All 30 patients included in the study completed the study hemodialysis procedure and were included in the analysis (Fig 1). Their characteristics and main hemodialysis procedure parameters are shown in Table 1. The main biochemical parameters before and after hemodialysis in both groups are shown in Table 2. The mean iCa values during dialysis in both groups are shown in Fig 2. Multilevel linear mixed effects regression analysis confirmed the designed difference between the groups, i.e. a statistically significant interaction effect of time and iCa target range group (coefficient for low iCa group -0.0006 (-0.0008 to -0.0003), p = <0.001), while the main effects of iCa target range group (coefficient for low iCa group 0.01 (-0.02–0.06), p = 0.661) and time (0.0002 (0.00006–0.0003), p = 0.079) were not significant. The mean iCa during dialysis in the normal iCa group was 1.12 ± 0.06 mmol/l, and in the low iCa group 1.06 ± 0.07 mmol/l (p <0.001), which was, on average, a little above the designed target range. This also resulted in different iCa, tCa and cCa after dialysis (see Table 2).

**Table 1:** Comparison of patient characteristics and hemodialysis procedure parameters in both groups. Data is shown as mean ± SD or percentage.

Parameter	normal iCa group	low iCa group	P value
N	15	15	/
age [years]	70 ± 16	62 ± 13	/
male gender	60%	87%	/
blood flow [ml/min]	250 ± 0	250 ± 0	1.000
mean 8% citrate infusion rate [ml/h]	150 ± 0	150 ± 0	1.000
mean 1M CaCl_2_ infusion rate [ml/h]	14.2 ± 1.0	12.3 ± 1.3	<0.001
dialysis duration [min]	246 ± 15	250 ± 22	0.559

**Table 2:** Comparison of laboratory values before and after hemodialysis procedures in both groups. Data is shown as mean ± SD.

parameter	normal iCa group	low iCa group	P value
iCa before [mmol/l]	1.15 ± 0.08	1.14 ± 0.11	/
iCa after [mmol/l]	1.14 ± 0.07	1.01 ± 0.05	<0.001
total calcium before [mmol/l]	2.14 ± 0.16	2.12 ± 0.19	/
total calcium after [mmol/l]	2.18 ± 0.22	1.95 ± 0.17	0.003
corrected calcium before [mmol/l]	2.29 ± 0.12	2.23 ± 0.25	/
corrected calcium after [mmol/l]	2.26 ± 0.20	1.97 ± 0.10	<0.001
magnesium before [mmol/l]	0.96 ± 0.15	0.92 ± 0.14	/
magnesium after [mmol/l]	0.86 ± 0.06	0.85 ± 0.09	0.691
pH before	7.35 ± 0.05	7.38 ± 0.05	/
pH after	7.39 ± 0.06	7.43 ± 0.03	0.040
bicarbonate before [mmol/l]	21.4 ± 2.9	22.8 ± 1.5	/
bicarbonate after [mmol/l]	23.1 ± 1.2	23.3 ± 1.3	0.782
iPTH before [pg/ml]	332 ± 241	378 ± 262	/
iPTH after [pg/ml]	368 ± 286	615 ± 415	0.068

**Fig 3:**
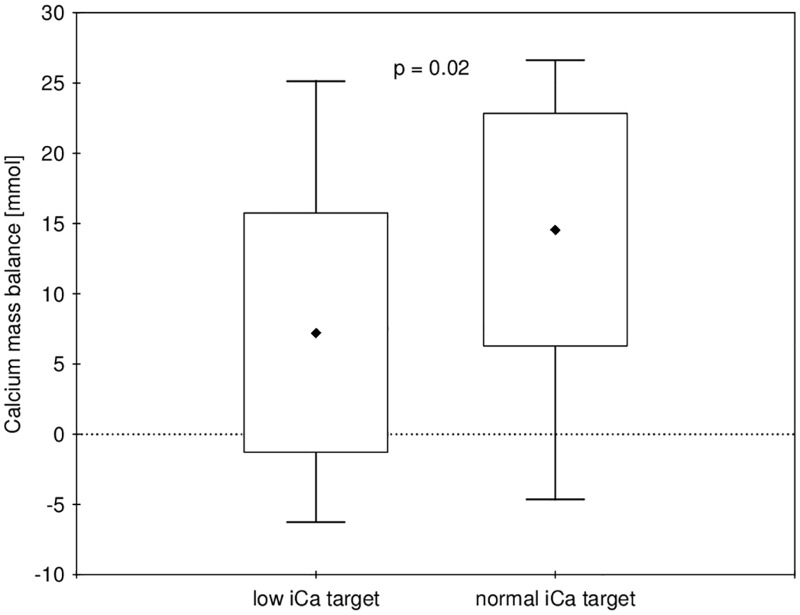
Comparison of calcium mass balance in both groups. Data is shown as mean ± SD and min-max.

**Fig 4:**
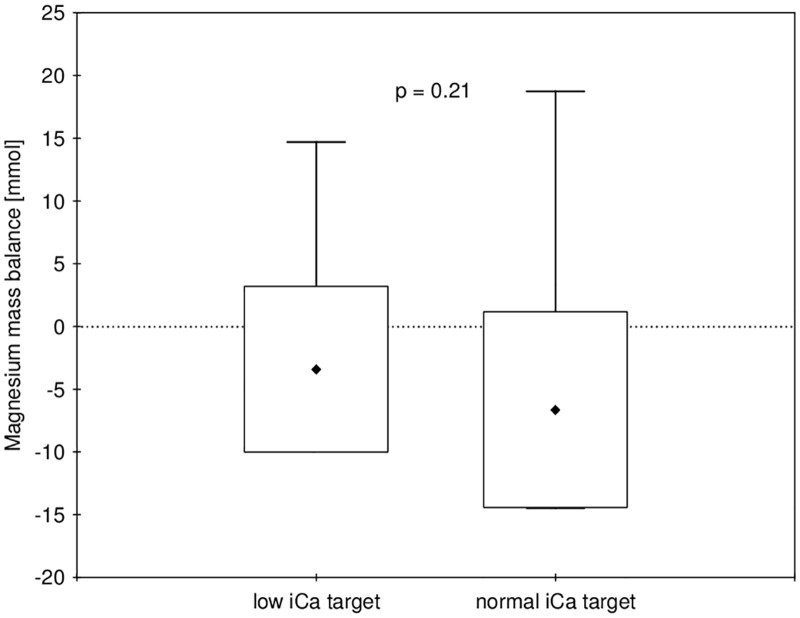
Comparison of magnesium mass balance in both groups. Data is shown as mean ± SD and min-max.

**Fig 5:**
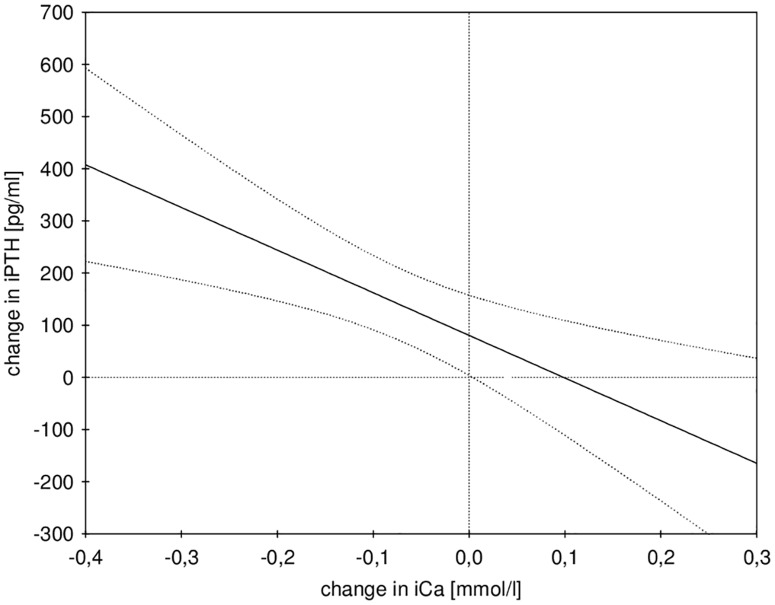
Linear regression model of changes in intact parathormone (iPTH) and ionized calcium (iCa) during the procedures (i.e. before vs. after) in all patients.

**Fig 6:**
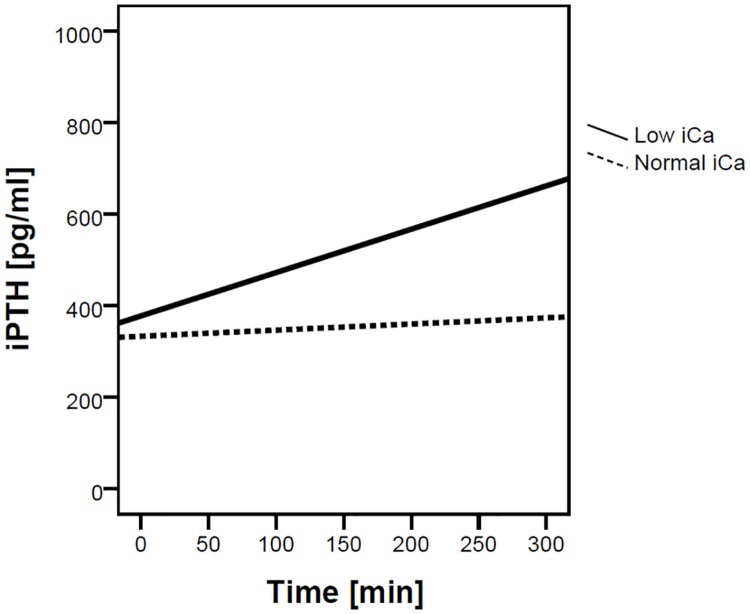
Interaction effect of time and the iCa target range group on iPTH.

Data on calcium and magnesium mass balance are presented in Table 3 and Figs 3 and 4. The mean delivered calcium during dialysis was 58.3±4.8 mmol in the normal and 51.5 ± 8.2 mmol in the low iCa group (p = 0.010), which resulted in a significantly higher mean positive calcium mass balance of 14.6 ± 8.3 mmol (i.e. 584 ± 333 mg) per procedure in the normal as compared to 7.2 ± 8.5 mmol (i.e. 290 ± 341 mg) in the low iCa group (p = 0.024). The values of iPTH before and after dialysis are given in Table 2. The change in iPTH during dialysis was significantly inversely related to the change in iCa (Beta = - 0.52, p = 0.003, see Fig 5). Multilevel linear mixed effects regression analysis (see Table 4) showed a statistically significant interaction effect of time and iCa target range group on iPTH, which is illustrated in Fig 6. In the low iCa target range group there was a statistically significant increase in iPTH as compared to normal iCa target range group (p = 0.008). The magnesium concentration in the spent dialysate was comparable in both groups (see Table 3) and resulted in a mildly negative magnesium mass balance during dialysis in both groups (-6.6 ± 7.8 mmol in the normal and -3.4 ± 6.6 mmol in the low iCa group (p = 0.206)). The multiple linear regression analysis of the factors, potentially affecting calcium mass balance, showed that in addition to the amount of infused calcium (p = 0.044) the calcium mass balance was independently correlated only to the iCa before dialysis (p = 0.003) (see Table 5).

**Table 3:** Parameters required for the calculation of calcium and magnesium mass balance in both groups. Data is shown as mean ± SD.

parameter	normal iCa group	low iCa group	P value
infused calcium [mmol]	58.3 ± 4.8	51.5 ± 8.2	0.010
total spent dialysate volume [l]	121 ± 9	124 ± 10	0.436
spent dialysate calcium [mmol/l]	0.362 ± 0.069	0.339 ± 0.054	0.882
spent dialysate magnesium [mmol/l]	0.554 ± 0.065	0.530 ± 0.047	0.244

**Table 4:** Relationship between iCa target range group and hemodialysis duration on iPTH (results of linear multilevel mixed effects model).

	coefficient (95% CI)	P value
intercept	329.8 (167.5–492.1)	< 0.001
low iCa target	45.0 (-184.4–274.5)	0.693
normal iCa target	ref.	
time	0.2 (-0.2–0.6)	0.413
low iCa target * time	0.8 (0.2–1.4)	0.008
normal iCa target * time	ref.	

**Table 5:** Multiple linear regression analysis of potential factors affecting calcium mass balance. Adjusted R^2^ for the whole model 0.54, p < 0.001.

variables	coefficient (Beta)	SE	P value
iCa before dialysis	-0.44	0.14	0.003
iCa after dialysis	0.16	0.18	0.398
iPTH before dialysis	-0.01	0.13	0.917
dialysis duration	-0.09	0.18	0.631
infused calcium	0.48	0.23	0.044

Visual assessment of the anticoagulation in the circuit was excellent at the arterial (median 5 (IQR 4–5)) and venous bubble trap (median 5 (IQR 5–5)), as well as the dialyzer (median 5 (IQR 5–5)). There was only one case of asymptomatic hypocalcemia (iCa <0.90 mmol/l) in the low iCa group, and none in the normal iCa group, which was not statistically significantly different (Yates corrected Chi square p = 1.000).

## Discussion

To our knowledge, this is the first study providing the measurement of calcium and also magnesium mass balance during hemodialysis with RCA in relation to the iCa target range. We have shown that even when a mild hypocalcemia is aimed for during citrate hemodialysis, this still results in a mildly positive calcium balance in many patients, whereas maintaining strictly normal iCa values results in a significantly more positive calcium balance. The magnesium balance is mildly negative during RCA hemodialysis with standard dialysate magnesium content.

What the desired calcium mass balance during a hemodialysis session is remains a matter of debate [15, 16], and depends also on the patient's oral calcium intake, especially with phosphate binders [25 – 27]. A neutral calcium balance during dialysis might be the best approach, advocated also by the guidelines [21]. There are many studies in literature reporting a calcium mass balance resulting from the use of different dialysate calcium levels in standard, i.e., heparin, dialysis [12 – 14]. In hemodialysis with RCA, the calcium balance has not yet been studied in detail, although RCA interferes with calcium more profoundly than heparin dialysis. Citrate exerts its anticoagulant effect by inducing severe hypocalcemia in the dialysis circuit, which is also maintained by the calcium-free dialysate. To prevent symptomatic hypocalcemia, calcium needs to be substituted by infusion, usually into the venous line. The calcium mass balance in RCA is therefore mainly dependent on the iCa levels maintained during citrate dialysis. A knowledge of the calcium mass balance during RCA is especially important when RCA is used for prolonged periods of time, or even permanently, in selected dialysis patients with persistent bleeding risk [8], or if RCA were to become the preferred mode of anticoagulation in chronic dialysis as well, as has already been the case in continuous methods.

In literature, we have found only two studies reporting the calcium balance in citrate hemodialysis. In a study by Kozik-Jaromin [18], which extensively analyzed citrate and calcium kinetics, the calcium balance was estimated (from calcium flux across the dialyzer) to be moderately positive (mean 5 mmol/session, maximum 17 mmol/session) when normal iCa was maintained during the procedure. On the other hand, the calcium balance was reported to be negative by 1.15 mmol/h of dialysis (i.e., approx. 4.6 mmol/4 h session) in another small study using total dialysate collection [28]. These results are more difficult to compare, since a very loose iCa target range (0.96–1.32 mmol/l) was used, and iCa before dialysis was not routinely measured (as part of the reduction in iCa measurements), although the average iCa after the sessions was moderately decreased (1.06 mmol/l) and somewhere between our two groups. Our study is the first to measure the calcium balance resulting from two different iCa target ranges. We observed a mildly positive calcium mass balance also when mild hypocalcemia was aimed for during citrate dialysis, and a markedly more positive balance when iCa was maintained within the normal range. There was considerable variation in calcium, as well as magnesium mass balance. For calcium this can partly be explained by the fact that mass balance was found to be independently correlated to pre-dialysis iCa and perhaps partly by the measurement imprecision (the dialysate calcium/magnesium concentration is multiplied by spent dialysate volume, which is over 100 litres, therefore multiplying the measurement error). A wide range of calcium mass balances are also reported for standard heparin dialysis with any given dialysate calcium concentration [12, 14, 26, 27].

Nevertheless, these results—including ours—do indicate that maintaining iCa within normal range probably results in a significantly positive calcium mass balance, accounting for approx. one third of the recommended total daily calcium intake with phosphate binders in hemodialysis patients [25]. In the long-term, continuously positive calcium balance (amounting to an additional calcium burden of over 90 g per year when our results are extrapolated) could be expected to significantly increase vascular calcifications, which are considered a significant contributor to the increased morbidity and mortality of dialysis patients [29, 30]. Multiple linear regression analysis showed that the only factor affecting calcium mass balance in addition to the amount of infused calcium (directly relating to iCa target range) was the patient's starting iCa value, which highlights the need to individualize treatment [31]. We therefore believe that allowing a mild hypocalcemia during RCA in chronic hemodialysis patients is a better strategy, especially when RCA is used for prolonged periods of time.

At first sight it might seem contradictory that positive calcium mass balance was measured also in the low iCa target group, although there was a drop in ionized as well as total calcium during dialysis. This is also not very supportive of the hypothesis that significant accumulation of citrate-calcium complexes during dialysis is responsible for a positive calcium balance, since this should be reflected by a high/normal calcium and increased total to ionized calcium ratio [32]. However, short-term calcium regulation is quite complex, and a rapidly exchangeable calcium pool thought to represent the bone surface was recently hypothesized [33], which is probably responsible for preventing severe calcium shifts with acute calcium loading or removal. It has already been observed that total as well as ionized calcium after dialysis do not correlate very well with calcium mass balance during dialysis [18]. Our results confirmed that ionized calcium after dialysis was not an independent predictor of calcium balance, but only ionized calcium before dialysis, in addition to the amount of calcium infused. Therefore, mildly reduced total calcium after dialysis might only reflect the negative calcium balance in the second half of the procedure, when iCa dropped significantly (see Fig 2). The calcium balance was probably positive in the first half of the dialysis procedure, when iCa was relatively normal, given the high variability of the pre-dialysis iCa values and their significant impact on calcium mass balance, resulting in a mildly positive overall balance in the low iCa target group.

Regarding the influence of iCa target range on iPTH, we have observed a significant increase in iPTH only in the low iCa target range. Overall, there was a negative correlation of the change in iCa during dialysis and the change in iPTH, which is a normal physiological response. It was previously shown in patients requiring acute hemodialysis with RCA that if mild hypocalcemia is allowed, iPTH rises and the change is predicted best by the change in iCa [34]. This increase is probably only transient, although it has not been studied in detail. We have previously reported in a small retrospective study that conversion to chronic RCA was a significant predictor of iPTH in the long term [8], although this might have been confounded by the natural progression of secondary hyperparathyroidism. In an era of calcimimetics, even a moderate long-term effect on iPTH would probably not be clinically relevant or unmanageable. Furthermore, an increase in iPTH might be desired in case of adynamic bone disease [35]. RCA with a patient-oriented calcium target range therefore allows for a more fine-tuned effect on iPTH as heparin dialysis with fixed dialysate calcium, although at three different concentrations.

The magnesium balance during hemodialysis has been studied in less detail. Maintaining magnesium levels within normal range is suggested [36], and dialysate with 0.5 mmol/l magnesium is usually used to achieve this. Calcium-free dialysate, used for RCA in the majority of protocols, also has the same magnesium content of 0.5 mmol/l. Magnesium, as a divalent cation, is also bound by citrate, and this could affect its mass balance in a complex way. Binding to citrate could increase the dialyzability of magnesium in the form of citrate-magnesium complexes, while on the other hand, the decrease in ionized magnesium could also increase magnesium flux from the dialysate. Our results show that a mildly negative magnesium balance was obtained regardless of the iCa target range, which resulted in a mild decrease in total magnesium, similar to heparin dialysis [36]. Although hypomagnesemia has recently been associated with increased mortality in dialysis patients [37], one should be aware that increasing the dialysate magnesium concentration in RCA to prevent a negative balance would probably require an increase in citrate dose, since both calcium and magnesium bind to citrate.

The strengths of our study, in addition to the randomized design, are the use of partial spent dialysate method for the measurement of mass balance, which is more precise than estimating balance from pre- and post-filter measurements, and the comparison of two iCa target ranges, which is relevant for clinical practice. There are also some limitations: the number of patients included is modest and due to the applied method of randomization (block randomization with block size 2 and lack of concealment), selection bias is rather likely to bias the study results. The bias effect could be estimated from the data at hand. Additionally, the target iCa was achieved slowly during the course of dialysis, although this is probably clinically appropriate, to prevent symptomatic hypocalcemia. Furthermore, we did not measure iPTH before the next dialysis to confirm the transient nature of iPTH increase, neither calcium nor magnesium after the completion of dialysis, to complete the picture of their dynamics.

In conclusion, we have demonstrated that maintaining strictly normal values of iCa during hemodialysis with RCA results in a significantly more positive calcium mass balance (584 mg of elemental calcium) as compared to a mild hypocalcemia (290 mg) during the procedure. Mild hypocalcemia, on the other hand, causes a significantly greater increase in iPTH, which is probably transient. To achieve a more neutral calcium balance during hemodialysis with RCA, we would recommend aiming for a mild hypocalcemia during hemodialysis with RCA, which did not result in a significant incidence of symptomatic hypocalcemia. This is especially important when RCA is used for prolonged periods in selected patients, e.g., those with persistent bleeding risk, and for the future design of automated RCA in chronic dialysis.

## Supporting Information

S1 CONSORT Checklist (DOC)

S1 File Raw study data. (XLS)

S2 File Original study protocol. (DOC)

S3 File Study protocol—English summary. (DOC)
