# Supplementary material for: Calcium Mass Balance during Citrate Hemodialysis: A Randomized Controlled Trial Comparing Normal and Low Ionized Calcium Target Ranges
Source: PLoS One. 2016 Dec 28;11(12):e0168593. doi: 10.1371/journal.pone.0168593 (PMC5193410; doi:10.1371/journal.pone.0168593)
Supplement: S2 File — (DOC) [file pone.0168593.s003.doc]

**Vloga za oceno primernosti raziskave**

**1. Naslov raziskave in financiranje:** "Nekatere izboljšave področne antikoagulacije s citratom". Raziskava bo opravljena v okviru doktorske naloge Nataše Lešević, dr. med. in raziskovalnega programa št. P3-0323 pri Javni agenciji za raziskovalno dejavnost RS, delno bo financirana tudi iz terciarnega programa 20110059 UKC Ljubljana; ne financira je nobena farmacevtska družba.

**2. Vodja raziskave in zdravnik, odgovoren za varnost oseb v raziskavi:**

doc. dr. Jakob Gubenšek, dr. med., specialist internist, Center za dializo, Klinični oddelek za nefrologijo, UKC Ljubljana, številka raziskovalca: 24053.

Sodelavci: Nataša Lešević, dr. med., prof. dr. Jadranka Buturović-Ponikvar, dr. med., prof. dr. Rafael Ponikvar, dr. med., Ratko Žugić, VZT

**3. Načrt, utemeljitev in protokol:**

***Uvod***

Standardno se za antikoagulacijo med hemodializo uporablja standardni heparin in nizkomolekularni heparini (1), ki imajo sistemski antikoagulacijski učinek. Pri bolnikih, pri katerih je sistemska antikoagulacija zaradi aktivne krvavitve, povečanega tveganja zanjo ali neželenih učinkov heparine (s heparinom povzročena trombocitopemija, HIT) kontraindicirana, lahko uporabljamo brezheparinsko dializo, kjer obstaja pomembno tveganje za neuspešno izpeljavo dializne procedure zaradi koagulacije zunajtelesnega obtoka ali pa uporabimo regionalno citratno antikoagulacijo (RCA) (1). RCA se izvaja z infuzijo citrata (2) v arterijski krak dializnega sistema, kjer veže kalcij iz plazme do te mere, da je koagulacija zadovoljivo upočasnjena. V dializatorju se večina kompleksa citrat-kalcij zdializira (3). Na venskem kraku dializnega sistema (preden se kri vrne v bolnika) dodajamo kalcij, tako da vzdržujemo normalno koncentracijo ioniziranega kalcija v plazmi. S citratno antikoagulacijo imamo veliko kliničnih in raziskovalnih izkušenj (4 - 7).

Pri nekaterih redkih dializnih bolnikih je zaradi narave bolezni (kronične mikrokrvavitve iz prebavil, ki pomembno poslabšujejo anemijo, HIT, ipd.) kontraindikacija za heparin (dolgo)trajna, zato se odločimo za dolgotrajno citratno antikoagulacijo (7 - 9). V primerih dolgotrajne uporabe postanejo pomembni še nekateri vidiki RCA. Ni povsem jasno, kakšna je optimalna ciljna vrednost ioniziranega kalcija (iCa) v arterijski krvi bolnika, ki naj se med dializo z RCA vzdržuje. Kadar namreč citratno antioagulacijo uporabljamo krajši čas je bistveno predvsem preprečevanje nevarne hipokalcemije med samo dializo. Pri dolgotrajni uporabi citratne antikoagulacije pa je potrebno razmišljati tudi o masni bilanci kalcija med samo proceduro (ki je odvisna od vzdrževanega iCa) in vplivu na sekundarni hiperparatiroidizem oz. mineralno-kostno bolezen (10 - 13). Pri kroničnih dializnih bolnikih je zaradi pospešene atero- in arterioskleroze pozitivna bilanca kalcija nezaželena (14). Kakšna je bilanca kalcija med hemodializo z RCA ni znano oz. še ni bilo izmerjeno. Tudi vpliv na mineralno-kostno bolezen oz. sekundarni hiperparatiroidizem ni podrobno raziskan. Opisujejo porast vrednosti intaktnega parathormona (iPTH) med posamezno proceduro (15). V naši retrospektivni raziskavi smo pravtako kratkoročno opisovali (statistično neznačilen) porast iPTH, dolgoročno pa neželen porast iPTH pri polovici bolnikov (7).

RCA postaja vedno bolj uporabljana metoda antikoagulacije, in se razmišlja tudi o njeni uporabi pri bolnikih ki nimajo povečanega tveganja za krvavitev. Podatki iz literature namreč kažejo, da pri RCA pride do manjše interakcije krvi z umetnim materialom (dializatorjem) kot pri heparinski dializi, saj so te interakcije večinoma odvisne od koncentracije kalcija v zunajtelesnem obtoku, ki je pri RCA precej nizka, in zato RCA poveča biokompatibilnost dialize (16 - 19). Sodobni dializni aparati že ponujajo module za avtomatsko izvedbo RCA. V literaturi je opisanih več različnih protokolov za RCA, ki uporabljajo različne odmerke citrata (2, 4, 20, 21). Pri različnih protokolih opisujejo tudi različne ocene parametrov biokompatibilnosti, ki jo ocenjujemo glede na stopnjo aktivacije koagulacije (22), komplementa (18, 22), degranulacije trombocitov (19, 21) in levkocitov (18, 19). Neposredne primerjave vpliva dveh odmerkov citrata na parametre biokompatibilnosti pa v dostopni literaturi nismo zasledili.

***Namen, bolniki in metode dela****:*

1. raziskava: Primerjava masne bilance kalcija pri dveh ciljnih območjih ioniziranega kalcija med RCA

V različnih objavljeni protokolih za citratno antikoagulacijo upoštevajo različne ciljne vrednosti arterijskega iCa: mi npr. dopuščamo blago hipokalcemijo (tarčni iCa 0,9 – 1,0 mmol/l) (4), nekateri pa striktno vzdržujejo normalne vrednosti iCa (1,1 – 1,2 mmol/l) (20). Ker optimalna vrednost iCa, ki naj se vzdržuje med RCA ni jasna, in ker le-ta vpliva tudi na bilanco kalcija med dializo želimo primerjati masno bilanco kalcija med pri dveh vrednostih ciljnega iCa med RCA. Preveriti želimo hipotezo, da je pri višjem ciljnem iCa bilanca kalcija pozitivna, pri nižjem pa nevtralna, in da pri višjem ciljnem iCa pride do manjšega porasta intaktnega parathormona (iPTH) med dializo.

*Bolniki*: odrasli kronični dializni bolniki, ki bodo zaradi povečanega tveganja za krvavitev potrebovali hemodializo z RCA.

*Metode*: Dializne postopke bomo randomizirali v dve skupini: a) skupina s ciljnim iCa med dializo 0,95-1,05 mmol/l in b) skupina s ciljnim iCa med dializo 1,1-1,2 mmol/l; v vsaki skupini bo 15 dializ. Med dializo bomo zbrali proporcionalni del dializata (23) ter izračunali masno bilanco kalcija (infundirani kalcij med dializo - kalcij odstranjen z dializatom) in citrata, kontrolirali bomo iPTH pred/po dializi in spremljali pojavnost morebitne hipokalcemije med HD. Ocenjujemo, da bomo za preiskave iz dializnega sistema (brez zbadanja bolnika) odvzeli 20 ml krvi / dializno proceduro. Natančen protokol prilagamo.

2. raziskava: Primerjava vpliva dveh ciljnih območij iCa pri dolgotrajni citratni HD na parametre mineralno-kostne bolezni

---

(deleted, this part of the study has not yet been performed)

---

3. raziskava: Primerjava dveh odmerkov citrata med RCA

---

(deleted, this part of the study has not yet been performed)

---

***Pričakovani rezultati in pomen***

1. in 2. raziskava: pričakujemo, da bo masna bilanca kalcija pri strategiji blage hipokalcemije nevtralna ali blago negativna, iPTH pa bo med posamezno proceduro porastel, blago pa bo porastel tudi v 2-mesečnem obdobju. Pri strategiji z vzdrževanjem normalne vrednosti iCa pa pričakujemo pozitivno kalcijevo bilanco in vzdrževanje ali padec vrednosti iPTH. Rezultati raziskave bodo pomagali določiti optimalno vrednosti arterijskega iCa (tako z vidika bilance kalcija kot tudi vliva na iPTH) med RCA za hemodializne bolnike, kar je posebno pomemebno za bolnike na dolgotrajni citratni antikoagulaciji in v primeru, da bomo RCA pričeli uporabljati pri večjem številu dializnih bolnikov.

3. raziskava:

---

(deleted, this part of the study has not yet been performed)

---

**4. Ocena etičnih vidikov raziskave**:

1. raziskava: Primerjava bilance kalcija pri dveh ciljnih območjih iCa med RCA

RCA je koristna in potrebna za vključene bolnike zaradi tveganja krvavitve, prinaša pa običajna in poznana tveganja, predvsem nevarnost simptomatske hipokalcemije z motnjami ritma, ki je zelo redek zaplet in je možna predvsem, če bi prišlo do tehnične napake (npr. infuzija kalcija ne bi tekla). Ker že sedaj v klinični praksi uporabljamo nižjega od obeh preizkušanih ciljnih območij za iCa, in ob tem praktično nimamo simptomatskih hipokalcemij, ne pričakujemo povečanega tveganja za hipokalcemijo. Obremenitev za vključene bolnike predstavlja predvsem nekoliko večji odvzem krvi zaradi potrebnih preiskav (cca. 20 ml / dializo), ki pa bo neboleč, saj kri jemljemo iz dializnega sistema, in ob rednem spremljanju hemograma in zdravljenju anemije z epoetinom ocenjujemo kot sprejemljiv. Rezultati raziskave bodo pomagali izboljšati protokol za to obliko dialize za bodoče bolnike.

2. raziskava: Primerjava vpliva dveh ciljnih območij iCa pri dolgotrajni citratni HD na parametre mineralno-kostne bolezni

---

(deleted, this part of the study has not yet been performed)

---

3. raziskava: Primerjava dveh odmerkov citrata med RCA

---

(deleted, this part of the study has not yet been performed)

---

**5. Povzetek v jeziku, razumljivem ljudem brez medicinske izobrazbe:**

Regionalna citratna antikoagulacija (RCA) predstavlja glavno obliko preprečevanja strjevanja krvi med hemodializnim postopkom pri bolnikih, pri katerih obstaja povečana nevarnost krvavitve, saj preprečuje strjevanje krvi le v zunajtelesnem sistemu in ne v celotnem telesu. Svoj učinek citrat doseže preko zniževanja koncentracije kalcija v zunajtelesnem obtoku, kar prepreči strjevanje krvi, pred vračanjem krvi v telo, pa se kalcij nadomešča z infuzijo. Med hemodializo z RCA lahko pride do znižane koncentracije kalcija v krvi bolnika in nekaterih drugih presnovnih motenj. Dializo bolniki običajno dobro prenašajo, kot izredno redek zaplet (predvsem v primeru tehnične napake) pa obstaja možnost hudega znižanja koncentracije kalcija v krvi, ki lahko privede do motenj srčnega ritma. Z RCA imamo v našem centru že 10-letne izkušnje, do sedaj smo jo izvajali brez večjih zapletov.

1. raziskava: Primerjava bilance kalcija pri dveh ciljnih območjih ioniziranega kalcija med RCA

V raziskavi želimo primerjati dve ciljni vrednosti ioniziranega kalcija pri citratni antikoagulaciji in raziskati vpliv na kalcijevo ravnovesje in odziv parathormona. To je pomembno za presnovo kosti in kalcifikacije mehkih tkiv, ki so povezane z srčno-žilnimi boleznimi, ki so pogoste pri bolnikih na dializi.

2. raziskava: Primerjava vpliva dveh ciljnih območij iCa pri dolgotrajni citratni HD na parametre mineralno-kostne bolezni

---

(deleted, this part of the study has not yet been performed)

---

3. raziskava: Primerjava dveh odmerkov citrata med citratno dializo

---

(deleted, this part of the study has not yet been performed)

---

**6. Skrb za varnost in koristi oseb v raziskavi.** Bolnike bomo skrbno dializirali in spremljali po izdelanih protokolih, kot je to običajno, njihove koristi ne bodo prizadete. V primeru pomembnih ali za bolnika ogrožujočih zapletov, ki bi bili posledica RCA, bomo RCA prekinili in nadaljevali z drugo obliko antikoagulacije ali dializo v celoti prekinili.

**7. Varovanje zaupnosti osebnih podatkov**. Podatki zbrani v raziskavi bodo del bolnikove zdravstvene dokumentacije in bodo kot taki tudi varovani in hranjeni v Centru za dializo KO za nefrologijo KC.

**8. Pregled literature:**

1. European Best Practice Guidelines Expert Group on Hemodialysis, European Renal Association. Section V. Chronic intermittent haemodialysis and prevention of clotting in the extracorporal system. Nephrol Dial Transplant. 2002; 17 Suppl 7: 63-71.

2. Pinnick RV, Wiegmann TB, Diederich DA. Regional citrate anticoagulation for hemodialysis in the patient at high risk for bleeding. N Engl J Med. 1983 Feb 3;308(5):258-61.

3. Kozik-Jaromin J, Nier V, Heemann U, Kreymann B, Böhler J. Citrate pharmacokinetics and calcium levels during high-flux dialysis with regional citrate anticoagulation. Nephrol Dial Transplant. 2009 Jul;24(7):2244-51.

4. Buturovic-Ponikvar J, Cerne S, Gubensek J, Ponikvar R. Regional citrate anticoagulation for hemodialysis: calcium-free vs. calcium containing dialysate - a randomized trial. Int J Artif Organs. 2008 May;31(5):418-24.

5. Gubensek J, Buturovic-Ponikvar J, Ponikvar R. Regional citrate anticoagulation for single-needle hemodialysis: a prospective clinical study. Blood Purif. 2007;25(5-6):454-6. Epub 2007 Nov 29.

6. Buturovic J, Gubensek J, Cerne D, Ponikvar R. Standard citrate versus sequential citrate/anticoagulant-free anticoagulation during hemodialysis: a randomized trial. Artif Organs. 2008 Jan;32(1):77-81.

7. Gubenšek J, Kovač J, Benedik M, Marn-Pernat A, Knap B, Ponikvar R, Buturović-Ponikvar J. Long-term citrate anticoagulation in chronic hemodialysis patients. Ther Apher Dial. 2011 Jun;15(3):278-82.

8. Unver B, Sunder-Plassmann G, Hörl WH, Apsner R. Long-term citrate anticoagulation for high-flux haemodialysis in a patient with heparin-induced thrombocytopenia type II. Acta Med Austriaca. 2002;29:146-8.

9. Wiegmann TB, MacDougall ML, Diederich DA. Long-term comparisons of citrate and heparin as anticoagulants for hemodialysis. Am J Kidney Dis. 1987;9:430-5.

10. Basile C, Libutti P, Di Turo AL, Vernaglione L, Casucci F, Losurdo N, Teutonico A, Lomonte C. Effect of dialysate calcium concentrations on parathyroid hormone and calcium balance during a single dialysis session using bicarbonate hemodialysis: a crossover clinical trial. Am J Kidney Dis. 2012 Jan;59(1):92-101.

11. Drüeke TB, Touam M. Calcium balance in haemodialysis--do not lower the dialysate calcium concentration too much (con part). Nephrol Dial Transplant. 2009 Oct;24(10):2990-3.

12. Klemmer PJ. Calcium loading, calcium accumulation, and associated cardiovascular risks in dialysis patients. Blood Purif. 2005;23 Suppl 1:12-9.

13. Argilés A, Mion CM. Calcium balance and intact PTH variations during haemodiafiltration. Nephrol Dial Transplant. 1995 Nov;10(11):2083-9.

14. LeBoeuf A, Mac-Way F, Utescu MS, De Serres SA, Douville P, Desmeules S, Lebel M, Agharazii M. Impact of dialysate calcium concentration on the progression of aortic stiffness in patients on haemodialysis. Nephrol Dial Transplant. 2011;26(11):3695-701.

15. Apsner R, Gruber D, Hörl WH, Sunder-Plassmann G. Parathyroid hormone secretion during citrate anticoagulated hemodialysis in acutely ill maintenance hemodialysis patients. Anesth Analg. 2004;99:1199-204.

16. Hofbauer R, Moser D, Frass M et al. Effect of anticoagulation on blood membrane interactions during hemodialysis. Kidney Int. 1999 Oct; 56(4): 1578-1583.

17. Gabutti L, Ferrari N, Mombelli G, Keller F, Marone C. The favorable effect of regional citrate anticoagulation on interleukin-1beta release is dissociated from both coagulation and complement activation. J Nephrol. 2004 Nov-Dec; 17(6): 819-825.

18. Bos JC, Grooteman MP, van Houte AJ, Schoorl M, van Limbeek J, Nube MJ. Low polymorphonuclear cell degranulation during citrate anticoagulation: a comparison between citrate and heparin dialysis. Nephrol Dial Transplant. 1997 Jul; 12(7): 1387-1393.

19. Gritters M, Grooteman MP, Schoorl M et al. Citrate anticoagulation abolishes degranulation of polymorphonuclear cells and platelets and reduces oxidative stress during haemodialysis. Nephrol Dial Transplant. 2006 Jan; 21(1): 153-159.

20. Apsner R, Buchmayer H, Gruber D, Sunder-Plassmann G. Citrate for long-term hemodialysis: prospective study of 1,009 consecutive high-flux treatments in 59 patients. Am J Kidney Dis. 2005;45:557-64.

21. Richtrova P, Rulcova K, Mares J, Reischig T. Evaluation of three different methods to prevent dialyzer clotting without causing systemic anticoagulation effect. Artif Organs. 2011 Jan;35(1):83-8.

22. Opatrný K, Richtrová P, Polanská K, Wirth J, Sefrna F, Brandl M, Falkenhagen D. Citrate anticoagulation control by ionized calcium levels does not prevent hemostasis and complement activation during hemodialysis. Artif Organs. 2007 Mar;31(3):200-7.

23. Ing TS, Yu AW, Wong FK, Rafiq M, Zhou FQ, Daugirdas JT. Collection of a representative fraction of total spent hemodialysate. Am J Kidney Dis. 1995 May;25(5):810-2.

**9. Izjava odgovornega raziskovalca.** Podpisani odgovorni raziskovalec potrjujem, da se bomo v raziskavi držali načel Helsinške deklaracije, Oviedske konvencije in načel slovenskega Kodeksa medicinske deontologije. Izjavljam, da ne obstaja navzkrižje interesov. Mojo usposobljenost lahko preverite na COBISS, moja številka raziskovalca je 24053.

**10. Izjava predstojnika ustanove je priložena.**

Ljubljana, 4. 3. 2013.

Nataša Lešević, dr. med.

doc. dr. Jakob Gubenšek, dr. med.

mentor, vodja raziskave

**RAZISKAVA: primerjava dveh ciljnih vrednosti iCa med HD HD 8**%

| PROTOKOL ZA CITRATNO DVOIGELNO **HEMODIALIZO** z **8% citratom** in **NIŽJIM (0,95 - 1,05 mmol/l) ciljnim iCa** |
| --- |

(*Protokol pripravila: doc. Jakob Gubenšek, dr. med., prof. Jadranka Buturović-Ponikvar, dr. med.* 1. 12. 2015)

Za citratno dvoigelno hemodializo po tem protokolu potrebujemo:

- **dializno raztopino brez kalcija**,
- **8% natrijev citrat** (8% trinatrijev citrat dihidrat) na črpalko, **1 M CaCl_2_** (1 molarni kalcijev klorid) na perfuzor,
- izjemoma ampule Ca Fresenius/glukonat (v primeru hude hipokalcemije).

DVOIGELNA CITRATNA HEMODIALIZA - protokol:

- **Dializator** z visoko prepustno sintetično membrano površine 1,3 - 1,8 m^2^.
- **8% citrat** (8% trinatrij citrat dihidrat ) se nastavi na arterijsko krvno linijo, pred arterijskim lovilcem in za odvzemnim mestom za krvne preiskave, v začetni dozi **150 ml/uro**, ob **krvnem pretoku 250 ml/min**. Doze citrata načeloma ne spreminjamo, razen če je iz kakršnegakoli razloga drugačen tudi pretok krvi, takrat dozo citrata lahko spremenimo sorazmerno s spremembo pretoka krvi.
- Pred vtokom citrata v art. linijo moramo imeti mesto za odvzem krvi za preiskave (podaljšek s petelinčkom).
- **1 M CaCl_2_** teče na perfuzor na vensko krvno linijo, za venskim lovilcem, v začetni dozi **13 ml/h**, nato hitrost infuzije prilagajamo (za ±1-2 ml/h) vrednosti ioniziranega kalcija **(ciljni iCa 0,95 - 1,05 mmol/l)**.
- **Koncentracija natrija v dializatu** naj bo standardno **138, bikarbonata** pa **28** (koncentraciji se lahko spreminjata glede na klinično stanje in laboratorijske izvide bolnika)
- Pri bolniku je potrebno spremljati **znake hipokalcemije**: mravljinčenje (prsti, usta), krči, slabost, hipotenzija.
- V primeru hude hipokalcemije z **iCa ≤ 0,85 oz. simptomatske hipokalcemije** bi aplicirali 1 ampulo Kalcij Fresseniusa (počasi i.v. - 10 minut) in povečali **1 M CaCl_2_** po navodilu dializnega zdravnika. Preveriti je potrebno, če vse infuzije (citrat, kalcij) tečejo kot je pravilno, razmisliti je potrebno tudi o zmanjšanju infuzije citrata in preveriti tudi, če je bikarbonat v raztopini ustrezno znižan.

**POZOR!!!**

- Za **SEKVENČNO ULTRAFILTRACIJO (SUF)** in **ENOIGELNO DIALIZO** imamo drug protokol !!!! (drugačni odmerki citrata in kalcija !!!)
- Pravtako se je potrebno **IZOGIBATI** daljšemu **BY-PASSU** med hemodializo. V primeru daljšega by-passa je potrebno **CITRAT ZMANJŠATI NA 100 ml/h, KALCIJEV KLORID zmanjšati na 8 ml/h,** po ponovni vzpostavitvi pretoka dializne raztopine pa ponovno dati citrat in kalcijev klorid v prejšnji dozi.

**Shema procedure:**

8%

CITRAT

150 ml/h

**1 M**

arterijski venski  **CaCl_2_**

lovilec lovilec **13 ml/h**

### DIALIZATOR

**iCa, Na, K,** iCa **pred dializatorjem Dializat: Ca 0 koagulacijski Qb = 250 ml/min**

**plinska** (za oceno antikoagulacije) **Na 138, HCO_3_ 28 čas**

**ODVZEMI KRVI ZA PREISKAVE:**

- Odvzemno mesto za **plinsko analizo, koncentracijo kalcija in natrija** je na arterijski liniji, pred vstopom citrata v sistem. Pred odvzemom krvi za kratek čas ustavimo infuzijo citrata in upočasnimo krvno črpalko. Za določitev ioniziranega kalcija zadošča 0,3 ml krvi. Po odvzemu ponovno sprožimo infuzijo citrata. Če je izvid kalcija nepričakovano nizek ali visok ponovimo odvzem, v primeru nejasnosti lahko vzamemo kri iz periferne vene.
- **Jemanje vzorcev iz katetra** (pred dializo): po tem, ko iz katetra aspiriramo raztopino s katero je bil kateter zaprt (citrat, heparin), aspiriramo še 20 ml krvi, šele nato odvzamemo vzorec, aspiriranih 20 ml krvi pa vrnemo bolniku.
- Za **določanje koagulacijskega časa na ploščici** odvzamemo kri na venski liniji med dializatorjem in lovilcem.
- Za **določanje ioniziranega kalcija pred dializatorjem** (za oceno antikoagulacije) odvzamemo iCa iz odvzemnega mesta na art. liniji, za infuzijo citrata in pred dializatorjem, citrat mora ob odvzemu normalno teči.

**POTEK CITRATNE DVOIGELNE hemodialize z 8% citratom in ciljnim iCa 0,95-1,05 mmol/l HD 8%**

Ime in priimek bolnika: _____________________________________________ Rojen: ___ ___ _____

Žilni pristop: jug. / subkl. / fem. kat. / AVF HD monitor: _____________  **Datum HD: _________**

1 lumenski / 2 lumenski levo / desno **Dializator**: FX 60 / FX 80 / PF 140H / PF 170H / _______

**Dializna raztopina**: D 227 / ______ (Ca 0), dodan 1 M KCl ______ ml **Natrij: 138** / ______ **Bikarbonat: 28** / _____

Trajanje HD: _____ ur, **BRUTO! UF** (celotna HD, prepiši iz dializ. aparata): _________ ml, TT pred: _____ kg

**!! POZOR NOV PROTOKOL !! 8% citrat 150 ml/h !! NE delati SUF ali SN !!!**

**!!! CILJNI iCa iz arterije je 0,95 - 1,05 mmol/l !!!**

| **PARAMETRI  dialize** | **ob priključitvi** | po 30' | po 1 h | (dodatne spremembe) | po 2 h | po 3 h | po 4 h | **željene vredn.** |
| --- | --- | --- | --- | --- | --- | --- | --- | --- |
| pretok krvi |  |  |  |  |  |  |  | 250 |
| **inf. 8% citrata (ml/h)** |  |  |  |  |  |  |  | 150 |
| **inf. 1M CaCl _2_ (ml/h)** |  |  |  |  |  |  |  | 15 |
| **iCa^2+^ pred dializatorjem!  (= za citratom)** |  |  |  |  |  |  |  | 0,2 - 0,4 |
| **PREISKAVE** | **pred HD** | **po 30'** | po 1 h | (dodatni odvzemi) | po 2 h | po 3 h | po 4 h | **pred odključ.** |
| **iCa^2+^** iz arterije! |  |  |  |  |  |  |  |  |
| Na^+^ (= pred citratom) |  |  |  |  |  |  |  |  |
| **K^+^** |  |  |  |  |  |  |  |  |
| pH |  |  |  |  |  |  |  |  |
| HC0_3_ |  |  |  |  |  |  |  |  |
| **ZAPLETI**  (mravljinčenje, krči, hipotenzija, cukanje katetra, Ca Fresenius i.v., subjektivne težave) | |  |  |  |  |  |  |  |

* iCa pred dializatorjem - kontroliramo zaradi ocene antikoagulacije (glej na prvi strani) HC0_3_ = bikarbonat

**POZOR!** pred dializo resetiraj celotni iztočeni volumen na perfuzorju (kalcij) na 0, **po dializi zabeleži TOČNO količino iztočenega kalcija, ki ga zabeleži perfuzor: _______________ ml !!!**

**POZOR pred in po dializi odvzamemo biokemično epruveto za iPTH, ki jo takoj damo na led!**

**Pripombe**, opis zapletov: _________________________________________________________________

______________________________________________________________________________________

**Po končani hemodializi je potrebno oceniti antikoagulacijo:**

1. Oceniti strdek v arterijskem in venskem lovilcu arterijski lovilec: _______ venski lovilec: ______

(ocena 5 – ni strdka v lovilcu, 4 - fibrinski obroč, 3 - majhen koagul, 2 - velik koagul, 1 - koagulacija sistema)

2. Prešteti koagulirane kapilare v dializatorju (obkroži): Ocena: 5: do 20 koaguliranih kapilar

4: 21-50 koaguliranih kapilar

3: 51-100 koaguliranih kapilar

Ime in podpis tehnika, ki je vodil proceduro: 2: > 100 koaguliranih kapilar

1: > 20% koaguliranih kapilar

__________________________________

**RAZISKAVA: primerjava dveh ciljnih vrednosti iCa med HD HD 8**%

| PROTOKOL ZA CITRATNO DVOIGELNO **HEMODIALIZO** s **8% citratom** in **VIŠJIM (1,1 - 1,2 mmol/l) ciljnim iCa** |
| --- |

(*Protokol pripravila: doc. Jakob Gubenšek, dr. med., prof. Jadranka Buturović-Ponikvar, dr. med.* 1. 12. 2015)

Za citratno dvoigelno hemodializo po tem protokolu potrebujemo:

- **dializno raztopino brez kalcija**,
- **8% natrijev citrat** (8% trinatrijev citrat dihidrat) na črpalko, **1 M CaCl_2_** (1 molarni kalcijev klorid) na perfuzor,
- izjemoma ampule Ca Fresenius/glukonat (v primeru hude hipokalcemije).

DVOIGELNA CITRATNA HEMODIALIZA - protokol:

- **Dializator** z visoko prepustno sintetično membrano površine 1,3 - 1,8 m^2^.
- **8% citrat** (8% trinatrij citrat dihidrat ) se nastavi na arterijsko krvno linijo, pred arterijskim lovilcem in za odvzemnim mestom za krvne preiskave, v začetni dozi **150 ml/uro**, ob **krvnem pretoku 250 ml/min**. Doze citrata načeloma ne spreminjamo, razen če je iz kakršnegakoli razloga drugačen tudi pretok krvi, takrat dozo citrata lahko spremenimo sorazmerno s spremembo pretoka krvi.
- Pred vtokom citrata v art. linijo moramo imeti mesto za odvzem krvi za preiskave (podaljšek s petelinčkom).
- **1 M CaCl_2_** teče na perfuzor na vensko krvno linijo, za venskim lovilcem, v začetni dozi **15 ml/h**, nato hitrost infuzije prilagajamo (za ±1-2 ml/h) vrednosti ioniziranega kalcija **(ciljni iCa 1,1 - 1,2 mmol/l)**.
- **Koncentracija natrija v dializatu** naj bo standardno **138, bikarbonata** pa **28** (koncentraciji se lahko spreminjata glede na klinično stanje in laboratorijske izvide bolnika)
- Pri bolniku je potrebno spremljati **znake hipokalcemije**: mravljinčenje (prsti, usta), krči, slabost, hipotenzija.
- V primeru hude hipokalcemije z **iCa ≤ 0,85 oz. simptomatske hipokalcemije** bi aplicirali 1 ampulo Kalcij Fresseniusa (počasi i.v. - 10 minut) in povečali **1 M CaCl_2_** po navodilu dializnega zdravnika. Preveriti je potrebno, če vse infuzije (citrat, kalcij) tečejo kot je pravilno, razmisliti je potrebno tudi o zmanjšanju infuzije citrata in preveriti tudi, če je bikarbonat v raztopini ustrezno znižan.

**POZOR!!!**

- Za **SEKVENČNO ULTRAFILTRACIJO (SUF)** in **ENOIGELNO DIALIZO** imamo drug protokol !!!! (drugačni odmerki citrata in kalcija !!!)
- Pravtako se je potrebno **IZOGIBATI** daljšemu **BY-PASSU** med hemodializo. V primeru daljšega by-passa je potrebno **CITRAT ZMANJŠATI NA 100 ml/h, KALCIJEV KLORID zmanjšati na 8 ml/h,** po ponovni vzpostavitvi pretoka dializne raztopine pa ponovno dati citrat in kalcijev klorid v prejšnji dozi.

**Shema procedure:**

8%

CITRAT

150 ml/h

**1 M**

arterijski venski  **CaCl_2_**

lovilec lovilec **15 ml/h**

### DIALIZATOR

**iCa, Na, K,** iCa **pred dializatorjem Dializat: Ca 0 koagulacijski Qb = 250 ml/min**

**plinska** (za oceno antikoagulacije) **Na 138, HCO_3_ 28 čas**

**ODVZEMI KRVI ZA PREISKAVE:**

- Odvzemno mesto za **plinsko analizo, koncentracijo kalcija in natrija** je na arterijski liniji, pred vstopom citrata v sistem. Pred odvzemom krvi za kratek čas ustavimo infuzijo citrata in upočasnimo krvno črpalko. Za določitev ioniziranega kalcija zadošča 0,3 ml krvi. Po odvzemu ponovno sprožimo infuzijo citrata. Če je izvid kalcija nepričakovano nizek ali visok ponovimo odvzem, v primeru nejasnosti lahko vzamemo kri iz periferne vene.
- **Jemanje vzorcev iz katetra** (pred dializo): po tem, ko iz katetra aspiriramo raztopino s katero je bil kateter zaprt (citrat, heparin), aspiriramo še 20 ml krvi, šele nato odvzamemo vzorec, aspiriranih 20 ml krvi pa vrnemo bolniku.
- Za **določanje koagulacijskega časa na ploščici** odvzamemo kri na venski liniji med dializatorjem in lovilcem.
- Za **določanje ioniziranega kalcija pred dializatorjem** (za oceno antikoagulacije) odvzamemo iCa iz odvzemnega mesta na art. liniji, za infuzijo citrata in pred dializatorjem, citrat mora ob odvzemu normalno teči.

**POTEK CITRATNE DVOIGELNE hemodialize s 8% citratom in ciljnim iCa 1,1-1,2 mmol/l HD 8%**

Ime in priimek bolnika: _____________________________________________ Rojen: ___ ___ _____

Žilni pristop: jug. / subkl. / fem. kat. / AVF HD monitor: _____________  **Datum HD: _________**

1 lumenski / 2 lumenski levo / desno **Dializator**: FX 60 / FX 80 / PF 140H / PF 170H / _______

**Dializna raztopina**: D 227 / ______ (Ca 0), dodan 1 M KCl ______ ml **Natrij: 138** / ______ **Bikarbonat: 28** / _____

Trajanje HD: _____ ur, **BRUTO! UF** (celotna HD, prepiši iz dializ. aparata): _________ ml, TT pred: _____ kg

**!! POZOR NOV PROTOKOL !! 8% citrat 150 ml/h !! NE delati SUF ali SN !!!**

**!!! CILJNI iCa iz arterije je 1,1 - 1,2 mmol/l !!!**

| **PARAMETRI  dialize** | **ob priključitvi** | po 30' | po 1 h | (dodatne spremembe) | po 2 h | po 3 h | po 4 h | **željene vredn.** |
| --- | --- | --- | --- | --- | --- | --- | --- | --- |
| pretok krvi |  |  |  |  |  |  |  | 250 |
| **inf. 8% citrata (ml/h)** |  |  |  |  |  |  |  | 150 |
| **inf. 1M CaCl _2_ (ml/h)** |  |  |  |  |  |  |  | 15 |
| **iCa^2+^ pred dializatorjem!  (= za citratom)** |  |  |  |  |  |  |  | 0,2 - 0,4 |
| **PREISKAVE** | **pred HD** | **po 30'** | po 1 h | (dodatni odvzemi) | po 2 h | po 3 h | po 4 h | **pred odključ.** |
| **iCa^2+^** iz arterije! |  |  |  |  |  |  |  |  |
| Na^+^ (= pred citratom) |  |  |  |  |  |  |  |  |
| **K^+^** |  |  |  |  |  |  |  |  |
| pH |  |  |  |  |  |  |  |  |
| HC0_3_ |  |  |  |  |  |  |  |  |
| **ZAPLETI**  (mravljinčenje, krči, hipotenzija, cukanje katetra, Ca Fresenius i.v., subjektivne težave) | |  |  |  |  |  |  |  |

* iCa pred dializatorjem - kontroliramo zaradi ocene antikoagulacije (glej na prvi strani) HC0_3_ = bikarbonat

**POZOR!** pred dializo resetiraj celotni iztočeni volumen na perfuzorju (kalcij) na 0, **po dializi zabeleži TOČNO količino iztočenega kalcija, ki ga zabeleži perfuzor: _______________ ml !!!**

**POZOR pred in po dializi odvzamemo biokemično epruveto za iPTH, ki jo takoj damo na led!**

**Pripombe**, opis zapletov: _________________________________________________________________

______________________________________________________________________________________

**Po končani hemodializi je potrebno oceniti antikoagulacijo:**

1. Oceniti strdek v arterijskem in venskem lovilcu arterijski lovilec: _______ venski lovilec: ______

(ocena 5 – ni strdka v lovilcu, 4 - fibrinski obroč, 3 - majhen koagul, 2 - velik koagul, 1 - koagulacija sistema)

2. Prešteti koagulirane kapilare v dializatorju (obkroži): Ocena: 5: do 20 koaguliranih kapilar

4: 21-50 koaguliranih kapilar

3: 51-100 koaguliranih kapilar

Ime in podpis tehnika, ki je vodil proceduro: 2: > 100 koaguliranih kapilar

1: > 20% koaguliranih kapilar

__________________________________

**NAVODILA ZA ZBIRANJE DELNEGA DIALIZATA MED DIALIZO**

Ime in priimek bolnika: ______________________________ datum HD: ___________

1. Dializo izvajamo na aparatu (sobe 1, 2, 5, B oddelek):

- **Gambro AK100/200**, in uporabimo prilagojeno cev, ki odvaja dializat iz dializatorja, in ima dodano odvzemno mesto! ali
- **Gambro Artis**, ki že ima odvzemno mesto na cevi, ki odvaja dializat iz dializatorja

2. na odvzemno mesto na cevi, ki odvaja dializat iz dializatorja, nastavimo infuzijski sistem, ki ga speljemo preko črpalke Alaris v prazno, zaprto 3-5 litrsko vrečko, ki se sicer uporablja za plazmaferezo.

3. po priključitvi bolnika in začetku dialize vklopimo črpalko, da med celotnim potekom dialize odvzema odtekajoči dializat s hitrostjo 5 ml/min, pred odključitvijo bolnika črpalko ugasnemo.

5. **po dializi zabeležimo**:

- točno **trajanje dialize** (ure, minute): _______ h _______ min
- preverimo, da je bil nastavljeni **pretok dializata 500 ml/min** DA / NE,
- zabeležimo (iz dializnega aparata) celotni **volumen porabljenega dializata**: _________ ml
- zabeležimo (iz dializnega aparata) točno **BRUTO ultrafiltracijo**: ___________ ml
- preverimo (na perfuzorju) točen **volumen infundiranega kalcija**: ________ , __ ml
- izračunamo celotni **volumen v vrečki zbranega dializata**:
  ure HD x hitrost črpalke: ________ (5 ml/min=300 ml/h) = _________ ml

6. odvzem vzorcev dializata:

- vrečko z zbranim dializatom (okrog 3 litre) dobro premešamo
- odvzamemo 3 vzorce dializata (po 5 ml) v epruveto brez dodatkov (posebne za študijo!, bel pokrovček), ki jih opremimo z podatki o bolniku in datumom dialize in jih damo zmrzniti v zmrzovalnik pri ionometru / timski mizi.

Jakob Gubenšek, dr. med.
