# Supplementary material for: Calcium Mass Balance during Citrate Hemodialysis: A Randomized Controlled Trial Comparing Normal and Low Ionized Calcium Target Ranges
Source: PLoS One. 2016 Dec 28;11(12):e0168593. doi: 10.1371/journal.pone.0168593 (PMC5193410; doi:10.1371/journal.pone.0168593)
Supplement: S3 File — (DOC) [file pone.0168593.s004.doc]

**Comparison of calcium mass balance during citrate hemodialysis with normal and low ionized calcium target ranges - a randomized controlled trial**

**Aim**

To compare calcium mass balance with normal and low iCa target range during hemodialysis with regional citrate anticoagulation (RCA). We hypothesize that normal iCa target range results in positive calcium mass balance and smaller increase in iPTH, while mild hypocalcemia results in neutral calcium mass balance and grater increase in iPTH.

**Patients**

30 adult chronic hemodialysis patients, requiring RCA due to increased bleeding risk.

**Methods**

An open-label parallel group RCT, comparing a normal (1.1-1.2 mmol/) and a low (0.95-1.05 mmol/l) iCa target range during a single study hemodialysis procedure with RCA.

RCA protocol: blood flow at 250 ml/min, use calcium-free, magnesium 0.50 mmol/l dialysate. Infusion of 8% trisodium citrate into the arterial line at 150 ml/min. Infusion of 1 M calcium chloride into the venous line, starting at 13 ml/min in the low iCa group and at 15 ml/min in the normal iCa group, and adjusting the infusion to achieve the desired iCa target range. Visual assessment of the clotting in dialysis circuit on a 1-5 semiquantitative scale.

Laboratory measurements: iCa before, after 30 min and then after each full hour of HD, and after HD. Intact parathormone, blood gas analysis, total and corrected calcium before and after dialysis. Partial spent dialysate will be collected as follows: an infusion pump is connected to the spent dialysate line and set at 300 ml/h, collecting a representative sample the total spent dialysate during entire HD. After HD mix the collected dialysate and collect 2 samples, freeze at -20 °C, until calcium is determined in a sample of collected dialysate.

Primary outcome: calcium mass balance, calculated as: delivered calcium during dialysis (recorded in ml from the perfusor) - estimated calcium loss (= measured dialysate calcium concentration x total spent dialysate volume). Total spent dialysate volume calculated as: volume of fresh dialysate (recorded by the hemodialysis machine) + volume of gross ultrafiltration + volume of collected dialysate

Secondary outcomes:

- safety endpoint: the occurrence of hypocalcemia during HD (iCa < 0.90 mmol/l)

- visual assessment of the anticoagulation in the circuit after dialysis on a semi-quantitative score of 1 (worst) to 5 (best).
